# Experimentation du virus charbonneux: “Le Pelerin,” 1922. Homage a Louis Pasteur. Dessin de Damblans.

**DOI:** 10.3201/eid0802.020200

**Published:** 2002-02

**Figure:**
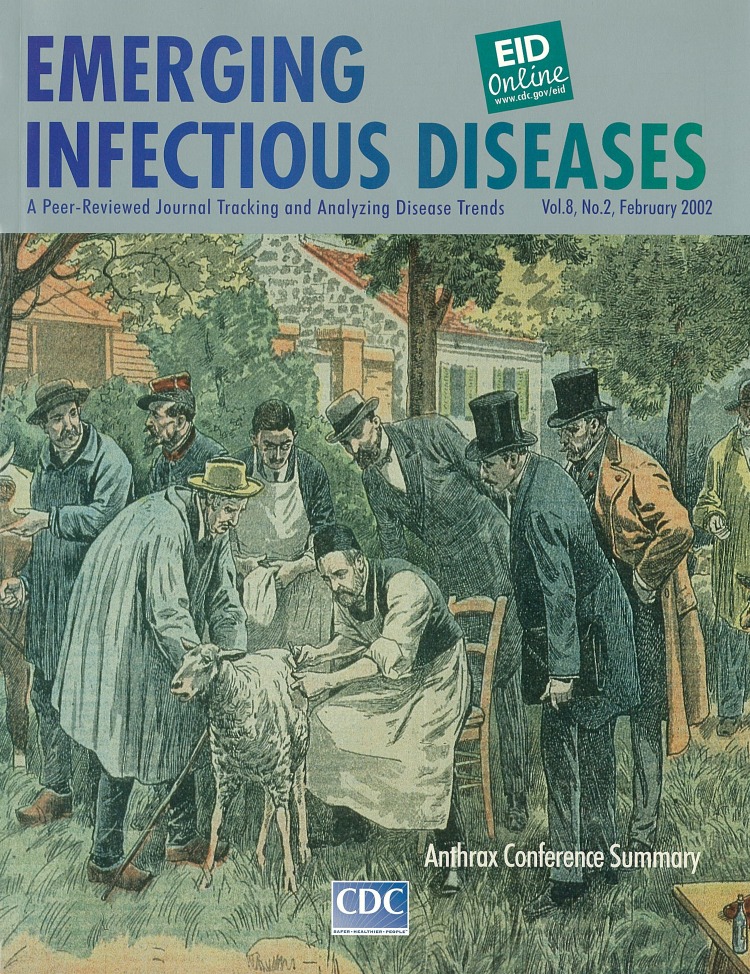
**Experimentation du virus charbonneux: “Le Pelerin,” 1922.** Homage a Louis Pasteur. Dessin de Damblans

In 1881, Louis Pasteur announced his theory that vaccinating livestock, in effect giving animals a disease, would protect them from that disease. Many contemporaries of Pasteur were skeptical about this radical idea as they were skeptical about the germ theory of disease. One of these contemporaries, the famous veterinarian Monsieur H. Rossignol, challenged Pasteur to test his theory in public by vaccinating animals on his farm at Pouilly-Le-Fort, a small village outside Paris. 

Rossignol’s challenge came early in Pasteur’s efforts to prove the germ theory of disease and develop vaccines. No vaccine had been tested yet outside the laboratory. The risk of having something go wrong with the experiment was very high. Pasteur, however, took the challenge, confident that what had worked with 14 sheep in the laboratory would work with 50 in the field. 

In early May, 25 animals at Rossignol’s farm were inoculated for “charbon,” a disease now known as anthrax. Another 25 received no vaccine. On May 31, all 50 animals were injected with a culture of very virulent anthrax. Within 2 days, a group of farmers, veterinarians, pharmacists, and agriculture officials gathered at Rossignol’s farm to observe the results of the experiment. The results were as Pasteur had anticipated: all 25 sheep that had not been inoculated had died; all 25 inoculated sheep were in perfect health.Those gathered at Pouilly-Le-Fort that day witnessed the first successful vaccine and the introduction of effective protection against anthrax. 

Anthrax, an ancient disease reported by Homer and Hippocrates as extremely deadly, killed thousands of animals each year. Pasteur’s vaccine provided not only protection against anthrax but also proof for the germ theory of disease. Protection against anthrax opened the door for vaccines against smallpox and other diseases. From Pasteur’s work emerged the disciplines of immunology and bacteriology, which eventually led to vaccination of millions of people and prevention of many diseases.

Abstracted from Hero for Our Time by Paul Trachtman, Smithsonian, January 2002.

